# Autonomic Nervous System and Cardiovascular System

**Published:** 2008-10

**213**

**Caffeine withdrawal retains anticataleptic activity but *Withania somnifera* withdrawal potentiates haloperidol-induced catalepsy in mice**

Dandge YB, Barhate Shrikant A, Kasture SB, M Mohan

**MGV’s Pharmacy College, Panchavati, Nashik 422003, India.**

**Introduction:**

The previous study showed that chronic treatment with *Withania somnifera* extract (WS) inhibited haloperidol-induced catalepsy. It is suggested that caffeine and WS may be useful adjuvant in pharmacotherapy of Parkinson’s disease.

**Methods:**

There are no studies on effect of haloperidol on mice withdrawn from caffeine or *Withania somnifera*. We therefore studied the effect of single administration of WS (30 or 100 mg/kg i.p.) and / or caffeine (3 mg/kg i.p.); and withdrawal from 6 days treatment with WS and / or caffeine, on haloperidol-induced catalepsy in albino mice.

**Results:**

Single administration of both WS and caffeine, used either alone or in combination significantly inhibited catalepsy. Mice withdrawn from caffeine significantly inhibited haloperidol-induced catalepsy, but mice withdrawn from WS showed increased catalepsy.

**Conclusion:**

The study indicated that withdrawal from WS does not retain anticataleptic activity and caffeine but not WS may be a good adjuvant in pharmacotherapy of Parkinson’s disease.

**214**

**Evaluation of *in-vitro* cardiotonic activity of some animal bile secretions**

Prasad N, Uday V, Praveen B, Murali G, Ramakrishna D, Prabhakara MC

**Kakatiya Univarsity, Warangal, India.**

Bile of a large number of animals, belonging to the classes Pisces, Reptilia, Aves and Mammalia are used in Unani medicine for the treatment of different ailments. Bile samples of ox, cow, goat and sheep were collected from the slaughter house by carefully isolating gall bladder and pressing out into small flask. Isolated and homozinized hearts of wistar rats of either sex weighing (200-250 gm) were used for Na+-K+ATP-ase inhibitory of bile. Cardiotonic screening of bile secretions *Per se* were carried out using Hypodynamic Frog’s Heart, and Na+-K+ATP-ase inhibitory activity using standard method. All selected bile secretions have cardiac stimulant and cardiotonic activity. This is further confirmed by Na^+^/K^+^ ATPase inhibitory action. Animal bile can well sustain the cardiotonic activity of digoxin when given in combination with bile secretions. In the present observations, ox, cow, goat and sheep bile *per se* inhibited Na^+^ / K^+^, ATPase activity on cardiac muscle and exhibited dose-dependant cardiotonic activity. Ox bile *Per se* had more Na^+^ / K^+^ ATPase inhibition and the order of cardiotonic activity was found to be ox > cow > goat > sheep. The results reflect that the activity of ox, cow, goat and sheep *Per se* were comparable and greater than that of digoxin. This study reveals that the bile secretion is not just for the emulsification of fats and lipids but may play a very important role in the vital functions of the body, which definitely requires proper further investigation.

**215**

**Pharmacodynamic interaction of garlic with propranolol in ischemia-reperfusion induced myocardial damage**

Khajuria KD^1^, AsdaqSMB^1^, Inamdar MN^2^

**^1^Krupanidhi College of Pharmacy, Bangalore, India, ^2^Al-Ameen college of Pharmacy, Bangalore, India.**

**Introduction:**

Garlic (*Allium sativum*) is widely recognized as agent for prevention and treatment of cardiovascular diseases. The present investigation was undertaken to demonstrate the protective effect of different doses of garlic and to determine its interaction with propranolol (PRO), during ischemia-reperfusion injury (IRI) induced damage to myocardium using isolated perfused rat heart preparation.

**Methods:**

Female albino rats were treated with GH at three different doses of 125; 250 and 500 mg/kg orally for 30 days and PRO (10 mg/kg, *p.o*.) was incorporated in the interactive groups during the last seven days of GH treatment. The hearts were excised and mounted on modified Langendorff setup and subjected to 15 min global no flow ischemia and reperfused for 15 min. Perfusates and heart tissue homogenate (HTH) were subjected for biochemical determination. Histopathological studies were carried out subsequently using hematoxylin and eosin stain.

**Results and Conclusion:**

Pretreatment of animals with PRO, GH-125 and GH-250 (either alone or in combination) provided significant protection to myocardium from IRI damage as indicated by significant decrease in LDH and CK-MB activities in perfusate and an increase in HTH. Similarly, the recovery (%) in developed tension and heart rate were significantly more in treated groups during post-ischemia when compared to control. Moreover, GH-250 either alone or with PRO showed significant increase in activities of antioxidant enzymes such as SOD and catalase in HTH. However, GH-500 failed to show cardioprotective effect when given alone or along with PRO. These biochemical findings were supported by changes in histopathological studies.

**216**

**Possible role of Janus kinase-2 in attenuated cardioprotective effect of ischemic preconditioning in hyperhomocysteinemic rat hearts**

Singh G, Rohilla A, Bhalla HS, Singh K, Singh M, Balakumar P

**ISF College of Pharmacy, Moga, India.**

The present study investigated the possible role of Janus kinase-2 (JAK-2) in hyperhomocysteinemia-induced attenuation of cardioprotective effect of ischemic preconditioning (IPC). Rats were administered L-methionine (1.7g/kg/day, *p.o*.) for 4 weeks to produce hyperhomocysteinemia. Isolated Langendorff’s perfused normal and hyperhomocysteinemic rat hearts were subjected to global ischemia for 30 min followed by reperfusion for 120 min. Myocardial infarct size was assessed macroscopically using triphenyltetrazolium chloride staining. Coronary effluent was analyzed for LDH and CK release to assess the extent of cardiac injury. Moreover, the oxidative stress in heart was assessed by measuring TBARS, superoxide anion generation and reduced form of glutathione. The ischemia-reperfusion (I/R) has been noted to induce oxidative stress and myocardial injury in normal and hyperhomocysteinemic rat hearts. The hyperhomocysteinemic rat hearts showed enhanced I/R-induced myocardial injury with high degree of oxidative stress as compared with normal rat hearts subjected to I/R. Four episodes of IPC (5 min each) afforded cardioprotection against I/R-induced myocardial injury in normal rat hearts. However, IPC mediated myocardial protection against I/Rinjury was abolished in hyperhomocysteinemic rat hearts. Treatment with Tyrphostin AG490 (5 *µ*M), a selective inhibitor of JAK-2 has not affected the cardioprotective effect of IPC in normal rat hearts, but its treatment markedly restored the cardioprotective potential of IPC in hyperhomocysteinemic rat hearts. Thus, it is suggested that the high degree of oxidative stress produced in hyperhomocysteinemic rat heart during reperfusion and consequent activation of JAK-2 may be responsible to abolish the cardioprotective potential of IPC against I/R induced myocardial injury.

**217**

**Minocycline with aspirin, a new therapeutic approach for the treatment of diabetic neuropathy in rats**

Veeranjaneyulu A, Bhatt LK

**School of Pharmacy and Technology Management, NMIMS University, Mumbai, India.**

**Introduction:**

Increased production of Matrix Metalloproteinase-2 (MMP-2) and Matrix Metalloproteinase-9 (MMP-9) in diabetes leads to degradation of extra cellular matrix in blood vessels and enhance complications of diabetes. Increased presence of cyclooxygenases further enhances levels of these MMPs.

**Methods:**

In the present study we have targeted MMP-2 and MMP-9 overactivation in diabetic neuropathy using a known MMP-2 and MMP-9 inhibitor, minocycline with a non-selective COX inhibitor aspirin. Streptozotocin (55 mg/kg, *i.p*.) induced diabetic neuropathy was carried out in male rats. Peripheral neuropathy was monitored by measuring the motor nerve conduction velocity (MNCV), and histopathology of the sciatic nerve. Hyperalgesia was measured by tail flick latency and hot plate latency. Three weeks after diabetes induction, rats were treated with minocycline (50 mg/kg, *p.o*.), aspirin (50 mg/ kg, *p.o*.), or minocycline plus aspirin for a period of three weeks.

**Results:**

Three week treatment with combination of minocycline plus aspirin showed significant improvement in MNCV, hot plate latency and tail flick latency (56.96 ± 4.20 m/s *P*>0.05, 6.78 ± 0.37 s *P*>0.05, 2.0 ± 0.01 s *P*>0.05 respectively) when compared with diabetic control (39.53 ± 1.96 m/s, 3.52 ± 0.20 s, 3.5 ± 0.24 s respectively). Nerve sections from minocycline plus aspirin pretreated group revealed less structural damage as compared to STZ-diabetic rats.

**Conclusion:**

Results of the present study suggest that minocycline in combination with aspirin prevents the development of experimental diabetic neuropathy in rats and can be a potential approach for the treatment.

**218**

**Effect of *Cassia tora* seed extract on lipid profile in cholesterol fed rabbits**

Dixit M^1^, Trivedi P C^2^, Gupta R^3^

**^1^L.B.S. College of Pharmacy, Jaipur, India; ^2^Department of Botany, University of Rajasthan, Jaipur, India; ^3^L.B.S. College of Pharmacy, Jaipur, India.**

Ethanolic extract (50%v/v) of ***Cassia tora*** seeds have been investigated for the protective effects against diet induced hyperlipidaemia in rabbits. Hyperlipidaemia was induced in rabbits by feeding atherogenic diet containing coconut oil, hydrogenated vegetable oil and cholesterol for 90 days. Rabbits were divided into three groups, each group comprising of six rabbits. Group I served as normal control, Group II (hyperlipidaemic control) was fed with atherogenic diet and cholesterol (400 mg/kg bodyweight /day/rabbit), Group III was concurrently fed with *Cassia tora* seed extract (500 mg/kg bodyweight /day/rabbit) along with atherogenic diet and cholesterol. Significant increase (*P*<0.05) in serum total cholesterol, LDL cholesterol, HDL cholesterol, triglycerides, Total Cholesterol: HDL ratio and LDL : HDL ratio was observed by feeding atherogenic diet and cholesterol to rabbits for 90 days. In-group III, simultaneous administration of *Cassia tora* seed extract along with atherogenic diet and cholesterol significantly (*P*<0.05) prevented the rise in serum total cholesterol (-59.91%), LDL cholesterol (-61.56%), HDL cholesterol (-53.75%) and triglycerides (-62.39%) level as compared to hyperlipidaemic control group. The decrease in total cholesterol: HDL ratio was found to be significant (*P*<0.05) in *Cassia tora* group as compared to hyperlipidaemic control group. There was non-significant (*P*=n.s.) decrease in LDL: HDL ratio as compared to hyperlipidaemic control group. *Cassia tora* treated rabbits showed significant (*P*<0.05) increase in faecal cholesterol as compared to hyperlipidaemic control group.

**219**

**Protective effect of *Hypericum hircinum* on doxorubicin induced cardiotoxicity in rats**

Bhagat CN, Shah SD, Mohan M, Kasture SB

**M.G.V’s Pharmacy College, Nasik 422003, India.**

**Introduction:**

Oxidative stress is the main factor in Doxorubicin (DOX) induced cardiotoxicity. The free radical scavenging effects of the *Hypericum hircinum* on DPPH (2, 2-diphenyl-1-picrylhydrazyl) was measured *in vitro*. The protective effect of *H.hircinum* on DOX induced cardiotoxicity was measured in rats.

**Materials and Methods:**

The aerial parts of *Hypericum hircinum* L. (Hypericaceae) were collected from Arzana Province, Sardenia, Italy. The plant was extracted with 1:1 of acetone-ethanol for 72 h. Laboratory breed Wistar albino rats of either sex weighing between 150-200 g, maintained under standard laboratory conditions of 25 ± 1°C, and photo period (12 hr dark/12 hr light) were used for the experiment. Wistar rats received either DOX (3 mg/kg, i.p) every other day or combination of *Hypericum hircinum* (100 mg/kg and 200 mg/kg, p.o.) and DOX or *H. hircinum* (200 mg/kg, p.o.) extract alone for 2 weeks. Cardiotoxicity was assessed by recording changes in ECG, heart rate and measuring the levels of cardiac marker enzymes LDH, CPK, GOT and the antioxidant enzymes- reduced glutathione (GSH), superoxide dismutase (SOD), and lipid peroxidative value (LPO) at the end of treatment schedule.

**Result:**

Treatment with *H. hircinum* (100 mg/kg and 200 mg/kg) significantly (*P*<0.05) decreased the levels of LPO and cardiac marker enzymes, increased the levels of GSH and SOD, reversed the changes in ECG and prevented the decrease in heart weight in DOX treated group.

**Conclusion:**

The results suggest that the *H. hircinum* has protective effect on Doxorubicin induced cardiotoxicity.

**220**

**Cardioprotective activity of ethanolic root extract of *Abutilon indicum* in isoproterenol induced myocardial infarction in male wistar rats**

Amaranth KR, Inamdar MN

**Al-Ameen College of Pharmacy, Bangalore, India.**

The ethanolic extract of the roots obtained from *Abutilon indicum* (Malvaceae) was evaluated for protection against Isoproterenol (150 mg/kg body wt, s.c) induced myocardial infarction in male Wistar rats. Isoproterenol induced rats showed significant elevation in the levels of serum marker enzymes such as Creatinine Kinase- MB, Lactate dehydrogenase (LDH), Aspartate transaminase (AST) and Alanine transaminase (ALT) with significantly increased lipid peroxides and significant decrease in antioxidant parameters viz., Super oxide dismutase (SOD), Catalase (CAT) and Glutathione peroxidase (GPx) in heart homogenate and also increased serum uric acid level. Oral pretreatment with ethanolic root extract of *Abutilon indicum* (100 mg/kg body wt) daily for a period of 28 days, reduced significantly the elevated serum marker enzymes and lipid peroxidation and elevated the levels of SOD, CAT and GPx in the heart homogenate and decreased serum uric acid level. Histopathological observation also revealed a marked protection by the extract in myocardial necrotic damage. Our results show that treatment with ethanolic root extract of *Abutilon indicum* (100 mg/kg body wt) was safe and highly effective in preventing cardiovascular dysfunction in rats, possibly due to antioxidant property as revealed by the amelioration of histopathological changes and biochemical markers of cardiac tissue damage. However, ethanolic root extract of *Abutilon indicum* (500 mg/kg body wt) was found to produce myocardial injury on its own and failed to reverse the Isoproterenol induced myocardial injury.

**221**

**Rottlerin restored the abrogated cardioprotective potential of ischemic preconditioning in hyperhomocysteinemic rat hearts**

Rohilla A, Singh G, Reddy J, Arora MK, Singh M, Balakumar P

**ISF College of Pharmacy, Moga, India.**

The present study is designed to investigate the possible role of protein kinase C-delta (PKC-delta) in hyperhomocysteinemiainduced attenuation of cardioprotective potential of ischemic preconditioning (IPC). Rats were administered L-methionine (1.7 g/ kg/day, p.o.) for 4 weeks to produce hyperhomocysteinemia. Isolated Langendorff perfused normal and hyperhomocysteinemic rat hearts were subjected to global ischemia for 30min followed by reperfusion for 120 min. Myocardial infarct size was assessed macroscopically using triphenyltetrazolium chloride staining. Coronary effluent was analyzed for LDH and CK release to assess the degree of cardiac injury. Moreover, the oxidative stress in heart was assessed by measuring lipid peroxidation and superoxide anion generation. The ischemia-reperfusion (I/R) was noted to produce myocardial injury and oxidative stress in normal and hyperhomocysteinemic rat hearts. In addition, the hyperhomocysteinemic rat hearts showed enhanced I/R-induced myocardial injury with high degree of oxidative stress as compared with normal rat hearts subjected to I/R. Four episodes of IPC (5 min each) afforded cardioprotection against I/R-induced myocardial injury in normal rat hearts. On the other hand, IPC mediated myocardial protection against I/R-injury was abolished in hyperhomocysteinemic rat hearts. Treatment with rottlerin (10 *µ*M), a selective inhibitor of PKC-delta did not affect the cardioprotective effects of IPC in normal rat hearts; but its treatment significantly restored the cardioprotective potentials of IPC in hyperhomocysteinemic rat hearts. The high degree of oxidative stress produced in hyperhomocysteinemic rat hearts during reperfusion may activate PKC-delta, which may be implicated in the observed paradoxically abrogated cardioprotective potential of IPC in hyperhomocysteinemic rat hearts.

**222**

**Interaction of propranolol with garlic in isoproterenol induced myocardial infarction in rat**

Asdaq SMB^1^, Inamdar MN^2^

**^1^Krupanidhi College of Pharmacy, Bangalore, India; ^2^Al-Ameen College of Pharmacy, Bangalore, India.**

**Introduction:**

Garlic (*Allium sativum*) is one of the herbs that are widely believed to hold promise as therapeutically effective medicament for cardiovascular diseases either in presence or absence of conventional cardioprotective drugs including propranolol (PRO). The present investigation was undertaken to demonstrate the effect of different doses of garlic homogenate (GH) and to determine its interaction with PRO in isoproterenol (ISO) induced myocardial damage in rats.

**Methods:**

Female wistar albino rats were treated with GH at three different doses of 125; 250 and 500 mg/kg orally for 30 days and PRO (10 mg/kg, *p.o*.) was incorporated in the interactive groups during the last seven days of GH treatment. Myocardial damage was induced by subcutaneous administration of ISO (150 mg/kg body weight) for 2 consecutive days. Blood was withdrawn from retroorbital route 48 hrs after the first dose of ISO and serum was separated. Heart tissue homogenate (HTH) of the excised heart was prepared. Both serum and HTH are used for biochemical estimations. Histopathological studies were carried out subsequently using hematoxylin and eosin stain.

**Results and Conclusion:**

The PRO and GH 250 mg/kg was found to ameliorate the effect of ISO on SOD, catalase and retained the activities of the diagnostic marker enzymes such as lactate dehydrogenase (LDH) and creatine phosphokinase isoenzyme (CKMB). Incorporation of PRO during GH treatment provided further protection to myocardium from injury. However, higher dose of GH alone or in presence of PRO failed to prevent ISO induced myocardial injury. Histopathological determinations confirmed biochemical findings

**223**

**Pattern of drug utilization in patients with acute ST segment elevation myocardial infarction in a tertiary care hospital**

Pradeepa R^1^, Anuradha HV^2^, Cuckoo Aiyappa^3^, MC Shivamurthy^4^

**M.S.Ramaiah Medical College and Teaching Hospital, Bangalore, India.**

**Introduction:**

Myocardial infarction is one of the most common diseases in the world, with diabetes, hypertension and smoking contributing as a significant risk factor. The objective of this study is to evaluate the drug utilization pattern and the age and sex distribution in patients with acute myocardial infarction.

**Methods:**

A retrospective drug use review was conducted between May to July 2008 and 198 patient prescriptions were analyzed.

**Results:**

Out of 198 patients, 151 were males and 47 were females. Prevalence of the disease in males was high between age group 45-55 years(24.8%) whereas in females it was between age group 55-65 years(0.08%). 36.4% of patients had associated hypertension and 27.3 % had associated diabetes mellitus. 46.96% patients had acute anterior wall infarction followed by 28.3% patients with acute inferior wall infarction and 18.7% with evolved myocardial infarction. Mean drug usage per patient was 9.9. The drugs prescribed could be grouped into antiplatelets, thrombolytic agents, opioids, antihypertensives, anticoagulants, hypolipidemics, hypoglycemic agents, antianginal, inotropes, antibiotics and diuretics. The most commonly used drugs were aspirin and clopidogrel (both 96.96%) followed by heparin (95.45%), atorvastatin (93.93%), ACE inhibitors/ARBs (81.8%) and streptokinase (70.2%). The other drugs used were nitrates (75.75%), PPIs (54%), beta blockers (45.4%), diuretics (41.91%), inotropes (33.38%), trimetazidine (27.27%) and benzodiazepines (23.23%).

**Conclusions:**

From the present study it can be concluded that, all patients received multiple drug therapy and antiplatelet agents were the most commonly prescribed group of drugs suggesting their important role in Myocardial infarction.

**224**

**Efficacy study of alcoholic extract of *Cyperus rotundus* on high-fat diet-induced non-alcoholic fatty liver disease in rats**

Patel P, Mengi S

**C U Shah College of Pharmacy, S N D T Womens University, Mumbai, India.**

**Aim:**

The main purpose of present study was to test the efficacies and explore the potential mechanism of action of the alcoholic extract of the roots of *Cyperus rotundus* (CR) on high-fat dietinduced non-alcoholic fatty liver disease (NAFLD).

**Materials and Methods:**

Rats were randomly divided into six groups, containing six rats each. One group was given normal diet, while other five groups were fed a high-fat diet. After fifteen days of high fat diet treatment four of the high-fat diet-fed groups were administered Sylamarin (50 mg/kg, p.o.), and alcoholic extract of CR at three dose levels (35, 70, 140 mg/kg, p.o.) respectively for another 30 consecutive days. Fasting blood glucose, total cholesterol, and triglycerides were measured. Serum levels of aminotransferases and alkaline phosphates were estimated. Liver tissues from each group were processed for histological evaluation and estimation of liver total lipid content.

**Results:**

High fat diet treatment resulted in significant rise in the serum lipid profile as well as liver enzymes as compared to pallet diet group. Treatment with alcoholic extract of *CR* at all the three dose levels markedly improved NAFLD, ameliorating dyslipidaemia and fat accumulation in the liver. Serum aminotransferases and alkaline phosphates levels were significantly reduced by alcoholic extract of *CR* at all the dose levels. Importantly, the histological evaluation of rat liver demonstrated that alcoholic extract of *CR* decreased lipid accumulation.

**Conclusion:**

The results suggest that alcoholic extract of *CR* may have potential clinical application in the treatment of NAFLD.

**225**

**Role of mammalian target of rapamycin and peroxisome proliferator-activated receptor-γ (ppar-γ) in experimental obesity**

Goyal M, Reddy BV, Balakumar P, Singh M

**I.S.F. College of Pharmacy, Moga-142001, India.**

Obesity is a complex disorder due to imbalance of energy intake and expenditure and subsequent accumulation of body fat. The present study has been designed to investigate the effect of rapamycin and pioglitazone on various parameters of experimental obesity in rats. The feeding of high fat diet (HFD) for six weeks was employed to produce obesity in wistar rats. The HFD is shown to increase anthropometric parameters such as body weight, BMI, lee index and body fat depots. Further HFD feeding produce hyperglycaemia and increase serum levels of TG, TC, LDL, VLDL and decrease serum HDL level. In addition, histological studies revealed that HFD after six weeks produced microvasicular and macrovasicular steatosis in liver and significant increase in size of adipose tissue. However, treatment with rapamycin (5mg/kg/day *p.o*., 2 weeks) significantly decrease HFD induced increase in anthropometric parameters, lipid levels and significant decrease in microvasicular and macrovasicular steatosis in liver and size of adipose tissue. Concurrent treatment of pioglitazone (10 mg/kg/day *p.o*., 2 weeks) along with rapamycin (5mg/kg/day *p.o*., 2 weeks) significantly reversed the effect of rapamycin on HFD induced increase in body weight, BMI, Lee index, body fat depots. Moreover, treatment with pioglitazone (10 mg/kg/day *p.o*, 2 weeks) and rapamycin (5 mg/ kg/day *p.o*, 2 weeks) in combination do not produce significant decrease in the microvasicular and macrovasicular steatosis in liver and size of adipose tissue. Thus we concluded that rapamycin decrease obesity through its action on mTOR.

**226**

**Heparinized polymer and sirolimus coated balloon expandable coronary stents in rabbit iliac artery - drug eluting stents and vascular response**

Mandal R

**Sahajanand Medical Technologies Pvt. Ltd, Surat, India.**

New Zealand white rabbits (n=40) received bilateral stents implantation in iliac arteries- randomized to bare metal stents (BMS), heparinized biodegradable polymer (Hepamer) coated stents with low-dose (low-dose DES; 0.45*µ*g per mm^2^), or highdose (high-dose DES; 1.4*µ*g per mm^2^) sirolimus, or Cypher stents. Stents were harvested at 14-, 28-, and 90-days. At 14-day implants were analyzed by scanning electron microscopy (SEM) and confocal microscopy following immunostaining for platelet endothelial cell adhesion molecule-1 (PECAM-1) to assess endothelialization, while at 28- and 90-day light microscopy was performed. At 14-days, > 85% of stent struts showed endothelial coverage in BMS (92.5±5.0%) and Hepamer stents (86.3±6.3%), while a dose dependent decrease in endothelialization was observed in low-dose (68.8±14.4%) and high-dose DES (47.5±28.4%) by SEM analysis. Cypher stents showed further delay in strut endothelial coverage (27.5±10.6%). PECAM-1 expression was significantly greater in BMS as compared to all other groups. At 28-days, mean neointimal thickness was greater in BMS (0.05±0.02mm) and Hepamer stents (0.06±0.03mm) as compared to low- (0.03±0.01mm), high-dose DES (0.03±0.01mm) and Cypher stents (0.02±0.00mm). Percentages struts with fibrin deposition was overall low except for Cypher stents (32±9%) which was significantly greater than BMS (4±3%, *P*=0.016). At 90-days, DES remained dose dependently efficacious in neointimal suppression (Hepamer; 0.04±0.03mm, Low-dose DES; 0.03±0.01mm, and high dose DES; 0.02±0.01mm, *P*=0.06) with minimal inflammation. Hepamer coated sirolimus eluting stents showed sustained efficacy up to 90-days without excessive fibrin deposition and inflammation. Endothelialization in low-dose DES were comparable to BMS, while high-dose DES and Cypher stents showed significantly less endothelial strut coverage.

**227**

**Cardiac action potentials: The electrophysiological tool for early drug safety profiling**

Bansal V, Banerjee J, Ray A, Bhatnagar PK, Varshney A

**Ranbaxy Research Laboratories, Gurgaon, India.**

Drugs, although primarily intended for their therapeutic benefits are often associated with certain side effects. These unwanted pharmacological actions could range from relatively mild and tolerable effects like nausea and vomiting to more severe and life threatening ones like cardiac arrhythmias, genetic mutations etc. These side effects associated with a drug often limit drug’s therapeutic use or demand a judicious intervention over its administration. QT prolongation is one of the major known liabilities and has led to the market withdrawal of many approved drugs like cisaperide and terfenadine. The drug regulatory bodies have therefore formulated the ICH S7B guidelines in their process of drug approval. These ICH guidelines have become the gold standard for all drug discovery groups worldwide; and in turn demand a thorough and complete preclinical cardiac safety profiling for all NCE’s. As per the ICH guidelines cardiac tissue preparations from guinea pigs and rabbits serve as valuable preclinical cardiac safety models. At Ranbaxy, we have successfully established these cardiac action potential assays in guinea pig and Rabbit papillary muscles, for the first time in Indian pharma R and D. These assays have been validated with a battery of tool compounds and have shown to match the published efficacy of the compounds tested. These assays provide active support for our in house research programs and serve as valuable safety screens for prioritizing new chemical entities that have druggable properties.

**228**

**Hypolipidemic effects of *Chlorophytum borivilianum* tubers**

Deore SL, Khadabadi SS, Baviskar BA, Wane Tushar, Jain Ritesh, Kendale Gitesh, Koshti Gaurav

**Govt. College of pharmacy, Kathora naka, Amravati-444604. (M.S.), India.**

The association of raised serum cholesterol with cardiovascular disease is well known. Many plant extract containing saponins claimed to have hypolipidemic activity and hence the present study was carried out to study the effect of ethanolic extract of *Chlorophytum borivilianum* tubers (CBT) to investigate the possible hypolipidemic effects of CBT on rats fed with a high-cholesterol diet. CBT exhibited potential hypolipidemic activity when compared with a standard dose of lovastatin. The rats were divided into six groups of ten animals each. First Control, second cholesterol control, the third, fourth and fifth groups were given cholesterol and CBT in the doses of 100, 200, 500 mg/kg respectively. The sixth group was of standard lovastatin (7.2 mg/kg) in addition to cholesterol. Serum cholesterol, triglycerides and HDL were measured on days 0, 10 and 20. The changes on the lipid levels of each group were statistically analyzed using Student’s‘t’ test. Treatment demonstrated dose dependent hypolipidemic activity at 100, 200 and 500 mg/kg dose levels by decreasing serum lipid. There was a significant decrease in serum cholesterol in both the lovastatin and the CBT treated groups.

**229**

**Pharmacological interventions to ameliorate diabetic-nephropathy in rats**

Jain A, Reddy BVK, Balakumar P, Singh M

**I.S.F. College of Pharmacy, Moga-142001, India.**

Diabetic nephropathy is characterized by thickening of basement membranes and messangial expansion with progression into glomerulosclerosis, tubular atrophy and interstitial fibrosis, leading to renal failure. *Morinda citrifolia* is known to possess antioxidant, anti-inflammatory and hypotensive properties. Fenofibrate is a selective PPAR-α agonist and possess lipid lowering activity and fosinopril, an ACE inhibitor. Diabetes is produced by single dose of streptozotocin (50 mg/kg *i.p*.) administrated to rats. Four weeks after streptozotocin injection, rats were randomly divided into eight groups, namely control rats, diabetic rats, noni *per se* and diabetic rats treated with fosinopril (40 mg/kg *p.o*.), diabetic rats are treated with fenofibrate (32 mg/kg *p.o*.) or extract of *Morinda citrifolia* (1, 2 and 4 g/kg, *p.o*.) for 4 weeks. Renal function is assessed by estimation of serum creatinine, blood urea nitrogen, and urine albumin excretion. Further, oxidative stress is assessed by estimating thiobarbituric acid reactive substances and renal reduced form of glutathione in diabetic kidney. After 8 weeks, diabetic rats exhibited renal dysfunction, as evidenced by reduced creatinine and urea clearance and proteinuria. However, treatment with *Morinda citrifolia*, fenofibrate and fosinopril significantly attenuated renal dysfunctions. It may be concluded that these agents significantly reduced due to inhibition of renal lipid accumulations.

**230**

**Role of Glucose – Insulin – Potassium infusion in patient of acute myocardial infarction**

Gandhi R

**Maharaja Agrasen Medical College, Agroha –Hisar, Haryana) India.**

**Objective:**

To study the effect of Glucose - Insulin - Potassium (GIK) infusion in patients of AMI.

**Materials and Methods:**

The present study was conducted on 50 patients (25 controls & 25 study group) of AMI admitted in PBM Hospital, Bikaner. 25 IU of regular insulin and 40 mmol of KCL were taken and injected into 500 ml bag of 25% glucose, rate of infusion was 1.5 ml/ kg/hr./24hr. The activity was assessed by measuring various electrolytes & various cardiovascular events were noted.

**Result:**

Glucose, K+ were measured at different hour.s baseline, 6+2 hours, 24+4hours. It was seen that in GIK group the glucose value were significantly raised during infusion period. At 24+4hours, the mean + SE was 7.64+ 0.49. The ‘*P*. value (0.004) was significant (<0.05) as compared to control group patients. The K+ value was also found to be raised 4.37+6.21 mmol/L at 24+4hours. The ‘*P*. value (0.020) was statistically significant. During the 7 days hospitalization period in the control group, 2 (8%) patients had cardiogenic shock and heart failure. Reinfarction occurred in 1(4%) patient. In study group only 1(4%) patient had heart failure. The Mortality rate was 12% (3 of 25) in the control group and 4% (1of 25) in patients who received GIK infusion.

**Conclusion:**

These observations suggest that (GIK) infusion in patients of AMI reduces the morbidity & mortality rate as compared to the usual care only. Hence these results provide a rationale for the apparent explanation of beneficial effects of GIK in treatment of AMI.

**231**

**Rosiglitazone, a PPARγ agonist, restores endothelial function in aorta of high fat fed streptozotocin induced diabetic rats**

Parmar A B, Patel CT, Patel Rameshvar K, Kanzariya NR, Patel MP, Patel NJ

**S.K. Patel College of Pharmaceutical Education and Research, Kherva, India.**

**Objective:**

Primary goal of this study is to find out the effect of rosiglitazone (a PPAR gamma agonist) treatment on blood pressure and endothelial function in high fat fed streptozotocin induced diabetic rats.

**Methods:**

Animals were fed with high fat diet for two weeks prior to streptozotocin injection. A single dose of streptozotocin (35 mg/kg, i.p.) was administered for induction of diabetes. Diabetic animals were also fed with the high fat diet till the experiment termination. Plasma glucose was measured at the end of every week to confirm consistent hyperglycemia. Rosiglitazone (5 mg/kg) treatment was given for six weeks to the diabetic animals. In case of in-vitro study different concentrations of rosiglitazone (10^-12^ M to 10^-6^ M) were pre-incubated 20-25 minutes. Blood pressures of the animals were measured at every week end by non invasive tail cuff method. After 7 weeks from STZ induction, spiral rat aortic preparation was used for endothelial function study, by taking CRCs of Phenylephrine (10^-11^ to 10^-5^) and Acetylcholine (10^-11^ to 10^-5^).

**Result:**

Rosiglitazone treatment reduced blood pressure with significant reduction in blood glucose, insulin, triglyceride and cholesterol levels. Pre-incubation with rosiglitazone (10^-12^ M to10^-6^M) shows concentration dependent increased in acetylcholine induced relaxation in diabetic rat thoracic aorta. Four week treatment of rosiglitazone (5mg/kg) also showed significant increase in acetylcholine induced relaxation in diabetic rat thoracic aorta.

**Conclusion:**

From present study it can be concluded that rosiglitazone treatment in high fat fed streptozotocin induced diabetic rats lowers blood pressure and restores endothelial function.

**232**

**Irbesartan an angiotensin II receptor antagonist protects focal cerebral ischemia in rats**

Akhtar M^1^, Pratap R^1^, Pillai KK^1^, Khanan R^1^, Islam F^2^

**Jamia Hamdard, New Delhi, India.**

Angiotensin receptor antagonists (AT II) and nonsteriodal antiinflammatory drugs have been shown to protect against focal cerebral ischemia in middle cerebral artery occluded (MCAO) rats. The present study was designed to test multiple doses of irbesartan (IRB) a selective AT_1_ receptor antagonist, aspirin (ASP) a nonsteriodal anti-inflammatory drug and the combination of both drugs in MCAO rats. Focal cerebral ischemia was induced by MCA occlusion lasting for 2 hours followed by reperfusion for 22 hours. IRB was administered intragastrically and ASP orally for 7 days before the induction of ischemia. After 24 hours of ischemia, behavioral parameters were assessed and then animals were immediately sacrificed for assessment of infarct volume and oxidative stress parameters. Locomotor activity and grip strength were improved in IRB and ASP treated rats. Infarct volume was reduced in both IRB and ASP pretreated rats as compared with MCAO rats. An elevation of thiobarbituric acid reactive substance (TBARS) and a reduction in glutathione (GSH), superoxide dismutase (SOD) and catalase were observed following MCAO. Pre-treatment of IRB and ASP showed the reduction in TBARS, elevation in glutathione, SOD and catalase levels as compared with MCAO rats. The protective effects of IRB, an angiotensin II (AT II) receptors antagonist having affinity for AT_1_ receptors, could be due to its neuroprotective and free radical scavenging properties in cerebral ischemia. Further more, the combination of IRB and ASP can be useful as an add-on therapy in reducing the severity of the neurological deficits in cerebral ischemia.

**233**

**Evaluation of cardiotonic activity of fruits of *Garcinia indica* choisy**

Gubbi SR, Patil PS, Shinde AJ, Jarag RJ

**Bharati Vidyapeeth college of Pharmacy, Near chitranagari, Kolhapur, (M.S) - 416013, India.**

The present study is to evaluate the cardiotonic activity of ripened and dried fruits of Garcinia indica (Guttiferae). It is necessary to screen nature based drugs which increase cardiac muscle contractility with broad therapeutic index as digitalis intoxication remains a common clinical problem. Traditionally Garcinia indica have been used in heart complaints. Hence, the present study is to evaluate the cardiotonic activity of ripened and dried fruits of Garcinia indica. The experimental protocol was approved from IAEC Bharati Vidyapeeth College of pharmacy, Kolhapur registred under CPCSEA. The Aqueous and alcoholic extracts were prepared and evaluated for identification of chemical constituents by performing chemical tests and TLC. The cardiotonic activity was performed by using isolated frog heart in situ preparation. Enzyme study such as Na+/K+ ATPase and Ca++ATPase, Mg++ATPase, were done on the heart tissue. Aspartase transaminase, Alanine transaminase, Lactate dehydrogenase, Creatine phosphokinase were estimated in the heart tissue and serum of albino rats after administering the extracts for 7 days. The aqueous extract produced significant positive ionotropic and chronotropic actions on frog heart. The positive ionotropic effect was selectively inhibited by nifedipine. A significant decrease in membrane Na+/K+ ATPase, Mg++ATPase and an increase in Ca++ATPase pointed the basis for the cardiotonic effect. The aqueous extract produced positive chronotropic and positive ionotropic effects which were antagonized by propranolol indicating that these might have been mediated through beta adrenergic receptors.

**234**

**Cardioprotective effects of tetrahydrocurcumin, on lipid peroxides and antioxidants in experimentally induced myocardial infarction in rats**

Manjunatha PM, Md. Sultan Ali^1^, K Sandip^1^, Goli Divakar^1^

**Acharya and B. M. Reddy College of Pharmacy, Soldevanahalli, Chikkabanavara Post, Bangalore- 560090, Karnataka, India.**

The present study was undertaken to evaluate the Cardioprotective potential of tetrahydrocurcumin (THC) in an *in-vivo* rat the ischemia – reperfusion (I/R) model of myocardial infarction (MI). Male wistar rats were divided into four groups, each group received saline *i.p*. (control MI/R group), vehicle control MI/R group *i.p*., and THC 5mg and 10mg *i.p*. injection respectively. On the experiment day, each groups were subjected to *in-vivo* rat model of acute ischemia for 30 minutes occlusion of left anterior descending coronary artery (LAD) and there after reperfusion for 4hrs. MI/R resulted in significant cardiac necrosis (41.26 ± 1.93; 30.46 ± 1.30), elevation in tissue and serum lipid peroxidation (129.95 ± 5.62, 19.08 ± 1.37; 101.91 ± 5.78, 16.74 ± 0.61), elevation in cardiac marker enzymes AST, ALT and decline in antioxidant status catalase, reduced glutathione in the normal control MI/R group and vehicle control MI/R group. Myocardial infarction produced after I/R was significantly reduced in tetrahydrocurcumin of the myocardial antioxidant status (*P*<0.001), infract size reduction (*P*<0.001) compared to control and vehicle control I/R group. Furthermore, I/R induced lipid peroxidation was significantly (*P*<0.001) reduced by tetrahydrocurcumin. Cardioprotective of treatment group were likely results from suppression of oxidative stress. Histopathological examination further confirmed the protective effect of tetrahydrocurcumin on the MI/R heart.

**235**

**Cardioprotective potential of myricetin in isoproterenol-induced myocardial infarction in wistar rats**

Dhage PN, Tiwari RV, M Mohan, Kasture VS, Kasture SB

**M.G.V’s Pharmacy College, Nashik-03, India.**

**Introduction:**

The rat model of ISO-induced myocardial necrosis has often been used to evaluate several cardiac dysfunctions. The work was aimed to study the cardioprotective potential of Myricetin in Isoproterenol induced myocardial infarction in Wistar rats.

**Materials and Methods:**

Wistar albino rats were divided into groups of five each. Group I received saline, group II received ISO (85 mg/kg s.c., at interval of 24h for 2 days), group III received myricetin (100 mg/kg, p.o., for 3 weeks), group IV received myricetin (300 mg/kg, p.o., for 3 weeks), group V received myricetin (100) and ISO and group VI received myricetin (300) and ISO. After 12 h following the last dose of ISO, ECG, heart rate and changes to vascular reactivity to various catecholamines were recorded using Powerlab 4SP (ADInstrument, Australia). The serum of animals of all the groups was assessed for their cardiac maker enzyme levels. The heart of animals of all the groups were excised, weighed, subjected to SOD and CAT measurements, histopathological and staining studies. CCRC of 5-HT was obtained using isolated rat fundus strip.

**Result:**

Pretreatment with myricetin for a period of 21 days significantly (*P*<0.05) inhibited the effects of ISO on heart rate, levels of LDH, CK, AST, SOD, CAT, vascular reactivity changes, and ECG patterns. CCRC of 5-HT was shifted towards right in rats treated with myricetin. Myricetin treated animals showed a lesser degree of cellular infiltration in histopathological studies.

**Conclusion:**

Myricetin has a potential to inhibit the cardiotoxic effect induced by ISO partly through its antioxidant properties.

**236**

**Renal effects of calcium channel blockers**

Khichi KK, Mittal SR, Mathur AK, Chouhan O, Ambwani S, Gehlot A

**Dr. S.N. Medical College, Jodhpur, Rajasthan, India.**

**Objective:**

The aim of present study is to evaluate diuretic potentiality of Nifedipine and other calcium channel blockers like Verapamil and Diltiazem and it is in response to complaint of polyurea by some of our patients of Nifedipine monotherapy (10 mg t.i.d. only).

**Methods:**

Twenty four hours output of urine was measured in three groups of eight rabbits each, before and during seven days oral therapy with Nifedipine, Verapamil and Diltiazem. During study animals were given the same diet and water intake and room temperature was also kept constant.

**Results:**

All the drugs caused significant (*P* 0.001) increase in mean daily output of urine. All the drugs decreased levels of serum Na and K levels. K levels were affected only by Diltiazem.

**Conclusion:**

1. Inhibition of tubular transport seems to be the main mechanism.
2. Diuretic activity may be attributed to preferential vessels of glomerular filtration due to dilatation of glomerural vessels.
3. By affecting intracellular cyclic AMP stores and over all cellural energy metabolism.

**237**

**Evaluation of DPJ 834, a newly synthesized compound for antihypertensive activity**

Singh R^1^, Krishan P^1^, Bodhankar SL^2^ Singh M^3^

**^1^Punjabi University, Patiala, Punjab, ^2^Bharati Vidhyapeeth Deemed University, Pune, Maharashtra, ^3^ISF College of Pharmacy, Moga, Punjab, India.**

**Introduction:**

Cardiovascular disease is the leading cause of premature death in developed and developing countries. High blood pressure is one of the most prevalent risk factor for cardiovascular disease and it has been extensively demonstrated that early screening and management of individuals with high blood pressure level leads to reduction in the occurrence of stroke and possibly myocardial infarction. So the present study was designed to investigate anti hypertensive activity of newly synthesized compound DPJ 834 in animals.

**Methods:**

Swiss albino mice and wistar rats were employed in the study. Antihypertensive activity was measured by using isoprenaline induced tachycardia in rats, fructose induced hypertension and chronic kidney ligation method. Mean arterial blood and mice ECG pressure was measured by using BIOPAC system.

**Results:**

Low doses of DPJ 834 (0.3 and 1.0 mg/kg) in mice ECG showed onset of action at about 5 min while at higher doses (3 mg/kg) produced immediate onset of action. Administration of DPJ 834 in isoprenaline induced tachycardia in rats produced hypotension and bradycardia. Similarly in fructose induced hypertension and kidney ligation models DPJ 834 significantly attenuated the increase in blood pressure.

**Conclusion:**

DPJ 834 significantly attenuated the increase in blood pressure in isoprenaline induced tachycardia in rats, fructose induced hypertension and chronic kidney ligation method.

**238**

**Effect of myricetin on blood pressure and metabolic alteration in fructose hypertensive rat**

Waghulde HB, Godse SP, M Mohan, Kasture SB

**M.G.V’s Pharmacy College, Nashik-03, India.**

**Objective:**

To evaluate the effect of the Myricetin on blood pressure and some metabolic parameters in the fructose hypertensive rat.

**Materials and Methods:**

Male Wistar albino rats (200-250g) were divided into 7 groups. Group I received vehicle, group II received Myricetin (100mg/kg, p.o., for 6 weeks), group III received Myricetin (300mg/kg, p. o., for 6 weeks), group IV received fructose solution (10% p. o. for 6weeks), group V received fructose solution (10% p. o. for 6weeks) and Myricetin (100mg/kg, p.o., for 6weeks), group VI received fructose solution (10% p. o. for 6weeks) and Myricetin (300mg/kg, p.o., for 6weeks), group VII received Fructose 10% p.o. for 6 weeks) and nifedipine(10 mg/kg/day, p.o. for 6 weeks). Body weight was monitored every week. After the treatment schedule vascular reactivity to various catecholamines (NA, Adr, PE, Ang- II, 5-HT,) were determined using Powerlab 4sp (AdInstrument, Australia). Cumulative Concentration Response curves (CCRC) of 5-HT and Ang-II were obtained using isolated rat fundus strip and isolated ascending colon respectively in rats of all the groups. Glucose, cholesterol, triglycerides, and insulin levels were measured in serum of rats of all groups at the end of the experiment.

**Result:**

A significant (*P*<0.05) decrease in mean blood pressure response to various catecholamines was observed in rats treated with myricetin. The cumulative concentration response curve (CCRC) of 5-HT and Ang-II was shifted towards right in rats treated with myricetin. Fructose-fed rats showed significant rise in blood pressure and a significant rise in glucose, cholesterol, triglycerides levels as compared to control. Treatment with myricetin reduced elevated serum glucose, cholesterol, triglycerides and insulin levels in fructose-fed rats.

**Conclusion:**

Thus myricetin ameliorated the vascular and metabolic effects in fructose hypertensive rat.

**239**

**Renoprotection by benazepril and telmisartan in diabetes: A histopathological study**

Singh J, Budhiraja S, Arora B^1^, Lal H^2^

**Department of Pharmacology Pathology^1^ and Biochemistry^2^, PGIMS, Rohtak- 124001, India.**

**Objective:**

To observe effects of benazepril {ACE Inhibitor} and telmisartan {Ang. II receptor antagonist} on kidney of STZ diabetic rats.

**Methods:**

Albino rats (250-300 g) of either sex were used. Diabetic nephropathy (DN) was induced by injecting STZ (50 mg/ Kg, iv). Animals were divided in to 4 groups of 10 rats each. Group I – Control, Group II-Diabetic control (STZ50 mg /kg, iv single dose). Group III-STZ + benazepril (5 mg /kg, po, 5 days prior to STZ and continued for further 16 weeks.).Groups IV –STZ + telmisartan (10 mg/ kg, po, 5days prior to STZand continued for 16 weeks). Results were confirmed biochemically (blood, urine sugar, blood urea, creatinine clearance, urinary protein) as wellas histopathologically. After 16 weeks histological study was performed by standard techniques. Paraffin sections of 5 *µ*m were cut and stained with hematoxylin, eosin, PAS and silver methanamine.

**Results:**

Biochemical parameters with benazepril andtelmisartan showed beneficial effects of these drugs. Telmisartan was somewhat more potent than benazepril. Histopathology of kidney from benazepril and telmisartan treated groups showed mild focal glomerular sclerosis, thickening of basement membrane and mild inflammatory changes. On the other hand, severe andgross changes were noted in the kidney sectionsof STZ diabetic rat. Most of the glomeruli showed diffuse sclerosis with large acellular nodules, swelling and vacuolization of epithelial cells. Fibrin caps were present in capillary basement membrane, segmental sclerosis, basement membrane thickening and increase mesangial matrix expantion were present in most of the sections Most tubules were atrophic Kidney section from normal rat showed normal glomeruli, renal tubules and blood vessels.

**Conclusion:**

Biochemical and histological studies with benazepril and telmisartan demonstrate that benazepril and telmisartan delays the progression of structural changes in the STZ diabetic kidney.

**240**

**The assessment of hypolipidaemic activity of two thiazolidin-4-one derivatives in high fat diet fed swiss albino mice**

Kishore A, Mor V, Kutty NG

**Manipal College of Pharmaceutical Sciences, Manipal, India.**

**Introduction:**

Research on thiazolidin-4-ones in our lab has resulted in the development of a few hypoglycaemic and anti-inflammatory molecules. Since inflammation coupled with dyslipidaemia and hyperglycaemia accelerates development of cardiovascular diseases, the current study involves the assessment of hypolipidaemic activity of two thiazolidinone derivatives, NAT3 and NAT4.

**Methods:**

Swiss albino mice were fed high fat diet and fructose for 20 days to induce hyperlipidaemia. Subsequently drugs were administered for 20 days while maintaining the same high-fat diet. The study groups NAT3 and NAT4 were compared with the standard nicotinic acid, hyperlipidaemic control and normal control groups. Drugs were administered in a dose of 100mg/kg. After 20 days the blood glucose and lipid levels of the groups were compared.

**Results:**

Twenty days of high fat diet (HFD) significantly increased body weight, serum glucose, cholesterol and triglyceride levels in mice. NAT3 significantly reduced body weight (3.42+0.93 %) and serum glucose (10.74+3.6 %) compared to HFD control. No significant difference was observed in cholesterol levels of treated and untreated hyperlipidaemic groups. Elevated serum triglyceride levels reduced significantly in NAT3 (12.3 + 7.1%), NAT4 (13.6+4.5%) and Nicotinic acid (19+6.2%) groups.

**Conclusion:**

It may be concluded that the NAT3 and NAT4 have modest hypolipidaemic activity. The fact that NAT3 can also reduce hyperglycaemia makes it a possible candidate for further modification to generate leads for syndrome X. The Thiazolidinone ring may have a role in these actions, through modulation of PPAR alpha or gamma. Further studies are required to confirm this hypothesis.

**241**

**Benfotiamine attenuates nicotine and uric acidinduced vascular endothelial dysfunction in rats**

Sharma R, Chakkarwar VA, Kaur J, Singh K, Reddy BVK, Singh M, Balakumar P

**ISF College of Pharmacy, Moga, India.**

The study has been designed to investigate the effect of benfotiamine, a thiamine derivative, in nicotine and uric acid-induced vascular endothelial dysfunction (VED) in rats. Nicotine (2 mg kg^−1^ day^−1^, i.p., 4 weeks) and uric acid (150 mg kg^−1^ day^−1^, i.p., 3 weeks) were administered to produce experimental VED. The development of VED was assessed by employing isolated aortic ring preparation and estimating serum and aortic concentration of nitrite/nitrate. Further, the integrity of vascular endothelium was assessed using the scanning electron microscopy of thoracic aorta. Moreover, the oxidative stress was assessed by estimating serum thiobarbituric acid reactive substances (TBARS) and aortic superoxide anion generation. The administration of nicotine and uric acid produced VED by impairing the integrity of vascular endothelium and subsequently decreasing serum and aortic concentration of nitrite/nitrate and attenuating acetylcholine-induced endothelium dependent relaxation. Further, nicotine and uric acid produced oxidative stress, which was assessed interms of increase in serum TBARS and aortic superoxide generation. However, treatment with benfotiamine (70 mg kg^−1^ day^−1^, p.o.) or atorvastatin (30 mg kg^−1^ day^−1^ p.o., a standard agent) markedly prevented nicotine and uric acid-induced VED and oxidative stress by improving the integrity of vascular endothelium, increasing the concentration of serum and aortic nitrite/nitrate, enhancing the acetylcholine-induced endothelium dependent relaxation and decreasing serum TBARS and aortic superoxide anion generation. Thus, it may be concluded that benfotiamine reduces the oxidative stress and consequently improves the integrity of vascular endothelium and enhances the generation of nitric oxide to prevent nicotine and uric acid-induced experimental VED.

**242**

**To evaluate the hypolipidemic action of ethanolic extract of *Asarum canadense* on experimental animal models**

Singh B, Das S

**Assam Medical College, Dibrugarh, India.**

**Introduction:**

Hypolipidemic drugs are those which lower the level of lipoprotein in blood. The plant *Asarum canadense* belongs to the family Aristolochiaceae and used in dysentery, digestive problem, cough and cold, sore throat, scarlet fever etc. it has also got antioxidant property.

**Methods:**

Ethanolic extract of leaves of *Asarum canadense* (EACL) was prepared by percolation method and subjected to oral toxicity testing using OECD guidelines. 30 albino rats were divided into 5 equal groups. Group 1 (normal control) received 3% gum acacia 10ml/kg/day, Group 2 (EACL) 250mg/kg/day with normal diet, group 3 received high fatty diet (vanaspati ghee and edible coconut oil 3:2) 10 ml/kg/day, Group 4 received both high fat diet and EACL 250 mg/kg/day and Group 5 (standard drug) received simvastatin 1.8 mg/kg/day. All drugs were given orally for 60 days and serum lipid parameters (Total cholesterol, Triglycerides, HDL and LDL) were estimated on 0, 30 and 60 days by drawing blood from retro-orbital sinus. Statistical analysis was done by using one way ANOVA followed by Dunnett’s test. *P*<0.05 was considered of statistical significance.

**Results:**

There was significant (*P*<0.05) hyperlipidemia in group-3 as compare to normal control. EACL and standard drug significantly (*P*<0.05) improved serum lipid parameters as compared to group-3, although the effect of EACL was less than the standard (*P*<0.05). Group-2 showed significant hypolipidemia (*P*<0.05) compared to normal control.

**Conclusion:**

As revealed by the study EACL has got significant anti-hyperlipidemic and hypolipidemic actions.

**243**

**Effects of 4-(2-alkylthio -1-benzyl -5-imidazolyl –dihydropyridines on the isolated rat colon and right atrium contractility**

Hadizadeh F, Fatehi-Hassanabad M, Fatehi-Hassanabad Z, Zandieh M

**Mashhad University of Medical Sciences, Mashhad, Iran.**

Dihydropyridines are known as widely used antihypertensive drugs, which exert their therapeutic effects mainly through modulation of calcium channels activity. Dihydropyridins with calcium channel blocking properties cause relaxation of smooth muscle. In order to provide a pharmacological profile for some newly synthesized dihydropyridines, we investigated their effects on the isolated rat colon segments and the isolated rat atrium contractility. The tested compounds include alkyl ester analogues of nifedipine (5a-d), in which the ortho-nitrophenyl group at position 4 is replaced by 2- methylthio-1-benzyl-5-imidazolyl substituent, and nifedipine as a positive control substance. The compounds showed similar effects to that of nifedipine on the isolated rat colon. The potency of these analogues with concentration range 10^−5^ to 10^−4^ M was much lower compared to potency of nifedipine which was effective at 10^−8^ to 10^−5^ M (*P*<0.01). However, unlike nifedipine, the test compounds exerted significant positive inotropic effect on the isolated rat atrium (*P*<0.01). Our observations suggest that these analogues of nifedipine selectively enhance contractility of heart muscle while causing relaxation of intestinal smooth muscle. These compounds may serve as valuable probes to develop novel dihydropyridines with dual smooth muscle relaxant effect and positive inotropic action in heart.

**244**

**To study hypolipidemic and antianginal effect of simvastatin in patients of coronary artery disease**

S Rani, Bajaj VK, Walia R, Gill BS

**Govt. Medical College, Patiala, India.**

**Objective:**

To study the effect of simvastatin in decreasing frequency of attacks of angina, in reversal of ischemic changes on ECG and on lipid profile in patients of Coronary artery disease (CAD).

**Materials and Methods:**

In this prospective open randomized controlled trial, 30 patients of CAD fulfilling the inclusion criteria were randomly allocated into 2 groups of 15 each. One group received conventional treatment (i.e. Nitrates, beta blockers and aspirin) and other group received simvastatin (10-20 mg) in addition to conventional treatment for 2 months.

**Results:**

In conventional group, mean values at baseline were triglyceride(TG) 221.33±33.35, total cholesterol(TC) 235.5±22.50, low density lipoprotein(LDL)183.67 ±20.87, high density lipoprotein(HDL) 38.14±3.6, frequency of attack of angina(FOA) 2±0.5 and after 8 weeks mean and percentage changes were TG (267±36, 21%), TC (260±17, 11%), LDL (200±14, 9%), HDL (37±2, −2%), and FOA was (2.2±0.45, 9%). In simvastatin group, mean values at baseline were TG (214±49), TC (251.8±26.30), LDL (201±29), HDL (38±3.5), FOA (2±0.65) and after 8 weeks, mean and percentage changes were TG (167±38,−22%), TC(201±13,−20%), LDL (160±14,−20%), HDL (40±3, 5%), and FOA was (1.53±0.63,−23%). E.C.G was done at each visit and no reversals of ischemic changes were seen after 8 weeks of simvastatin treatment.

**Conclusion:**

Lipid lowering and decrease in FOA were statistically significant in simvastatin group at 2 month. But reversal of ischemic changes on ECG was not significant. Thus simvastatin may decrease the morbidity in patients of CAD on long term use.

**245**

**Role of GLP-1 in amelioration of hyperhomocysteinemia-induced vascular endothelial dysfunction**

Goyal SK^1^, Bijjem KV^2^, Singh M^2^

**^1^Department of Pharmacology, S.D. College of Pharmacy, Barnala- 148101, Punjab, India; ^2^Department of Pharmacology, I.S.F. College of Pharmacy, Moga-142 001, Punjab, India.**

**Objective:**

Glucagon like-peptide-1 (GLP-1) agonist, Exendin-4 has shown relaxant effect on rat conduit arteries in previous reports. It may be suggested that GLP-1 has role in vascular endothelial dysfunction. So, the present study had been designed to investigate the role of GLP-1 in hyperhomocysteinemia-induced vascular endothelial dysfunction in wistar rats.

**Materials and Methods:**

L-methionine (1.7% w/w, 4 weeks) was mixed with standard chow diet and administered to produce hyperhomocysteinemia. The vascular endothelial dysfunction was assessed by acetylcholineinduced endothelium dependent relaxations using isolated aortic ring preparation and estimation of serum concentration of nitrite/nitrate using Griess reagent. Moreover, oxidative stress was assessed by estimating serum thiobarbituric acid reactive substances (TBARS).

**Results:**

Administration of L-methionine resulted in significant development of hyperhomocysteinemia and vascular endothelial dysfunction was confirmed by significant attenuation of acetylcholine-induced endothelium dependent relaxations, reduced serum nitrite/nitrate concentration and increased serum TBARS as compared to sham control group. However, treatment with exendin-4 (1 microgram kg^−1^ day^−1^) or atorvastatin (30 mg kg^−1^ day^−1^, a standard agent) markedly ameliorated hyperhomocysteinemia-induced vascular endothelial dyfunction.

**Conclusion:**

It may be concluded that exendin-4 may have ameliorated hyperhomocysteinemia-induced vascular endothelial dysfuntion due to activation of GLP-1.

**246**

**Pharmacodynamic interaction of garlic with captopril in ischemia-reperfusion induced myocardial injury in rats**

Rao GS^1^, Asdaq SMB^1^, Inamdar MN^2^, Premkumar N^1^

**^1^Krupanidhi college of pharmacy, Bangalore, India, ^2^Al-Ameen college of Pharmacy, Bangalore, India.**

**Introduction:**

Garlic (*Allium sativum*) and its preparations have been widely recognized as agents for the prevention and treatment of cardiovascular diseases. Without scientific validation, simultaneous use of garlic with conventional antihypertensive such as captopril (CAP) is commonly observed. Hence, present investigation was undertaken to demonstrate the expected interaction of garlic homogenate (GH) with CAP during ischemia-reperfusion injury (IRI) induced damage to myocardium using isolated rat heart preparation.

**Methods:**

Female albino rats were treated with GH at three different doses of 125; 250 and 500 mg/kg orally for 30 days and CAP (30 mg/kg, *p.o*.) was incorporated in the interactive groups during the last seven days of GH treatment. The excised hearts were mounted on modified Langendorff setup and subjected to 15 min global no flow ischemia. Perfusates and heart tissue homogenate (HTH) were subjected for biochemical estimations and histopathological examination.

**Results and Conclusion:**

Prophylactic administration of CAP and GH-250 (either alone or in combination) provided significant protection to myocardium from IRI damage as indicated by significant decrease in LDH and CK-MB activities in perfusate and an increase in HTH. Similarly, the (%) recovery in developed tension and heart rate were significantly more in treated groups during post-ischemia when compared to IRI control. Moreover, GH-250 either alone or with CAP showed significant increase in activities of antioxidant enzymes such as superoxide dismutase (SOD) and catalase in HTH. However, GH- 500 failed to provide recovery to myocardium from IRI in presence or absence of CAP. These biochemical findings were supported by histopathological observations.

**247**

**Reversal of altered vascular reactivity and vascular structure in diabetic rats by the combination of minocycline and aspirin**

Bhatt LK, Veeranjaneyulu A

**School of Pharmacy and Technology Management, NMIMS University, Mumbai, India.**

**Introduction:**

Strategies that interrupt the Matrix Metalloproteinase-2 (MMP-2) and Matrix Metalloproteinase-9 (MMP-9) system have been shown to reduce the ensuing threatening risk factors of vascular complications of diabetes by alteration in Extracellular Matrix (ECM).

**Methods:**

The present study was carried out to investigate the combined effect of chronic treatment of minocycline, a known MMP-2 and MMP-9 inhibitor with a non-selective COX inhibitor aspirin on the aortic reactivity of streptozotocin (STZ 55 mg/kg *i.p*.) diabetic rats. Contractile response to phenylephrine (10^−5^ M) and relaxation responses to acetylcholine (10^−9^ to 10^−4^ M) were obtained from aortic rings of diabetic rats. Histology of aorta was done by using Van-Gieson’s differential stain method and collagen level was estimated by image analysis.

**Results:**

Contraction of aortic strips from minocycline plus aspirin treated diabetic rats to phenylephrine was attenuated as compared to vehicle-treated diabetic rats. In addition, endothelium-dependent relaxation responses induced by acetylcholine were significantly higher (pD2 = 6.44 ± 0.24 and % relaxation 27.84 ± 2.537, *P* < 0.05) in treated rats as compared to diabetic rats (pD2 = 5.45 ± 0.18 and % relaxation 59.64 ± 2.99). Image analysis of aortic slides revealed that collagen level was significantly decreased in combined treated group when compared with diabetic control.

**Conclusion:**

Results of the present study suggest that minocycline in combination with aspirin reverse the endothelial dysfunction and normalize the endothelium-dependent relaxation responses in STZ-diabetic rats by reducing collagen level in ECM and can be a potential approach for the treatment.

**248**

**A study of prescribing pattern for antihypertensive drugs, rationality of prescriptions and patient awareness in a tertiary care center**

Shetty Y, Nigade J, Salagre S, Bichile L, Rege N

**Seth G.S. Medical College and K.E.M. Hospital, Mumbai, India.**

**Objective:**

To study the prescribing patterns, rationality of the prescription and awareness of patients about their prescriptions.

**Methods:**

After taking Ethics Committee approval, from May 2006 to March 2007, prescriptions of hypertensive patients who were prescribed antihypertensive drugs within the preceding 6 months by physicians and attending the Hypertension OPD of the institute for first time were reviewed using a prevalidated performa. Patients’ awareness regarding medications and disease was assessed using a questionnaire, the answers were scored. Data was analyzed using descriptive statistical analysis.

**Results:**

Review of 102 prescriptions revealed that per prescription 1.09 ± 0.89 antihypertensive drugs were prescribed; CCB being the commonest (56.5%). 98% prescriptions were incomplete with respect to dose, dosage form, frequency, duration and only 19 had stated some form of non-pharmacological therapy. Blood pressure levels at the time of prescription were not mentioned in 25 prescriptions. In 4 prescriptions, either the drugs were not suitable for compelling indication or agents from same group were combined. Awareness about the disease and its complication was absent in 70% of patients. Only, 65% of patients knew about the number of anti-hypertensive drugs prescribed and of these 94% knew the correct way of drug intake. Compliance was excellent in 53%.

**Conclusion:**

The number of irrational prescriptions and poor awareness level amongst patients about their disease and drugs prescribed as detected in the present study indicate a need for an educational intervention, targeting physicians and patients to improve the quality of health care at present.

**249**

**Synergism and additive interactions of combination of forskolin with anti-hypertensive agents on isolated rat thoracic aorta in normal and diabetic rats**

Bondal SS, Saraf MN

**Bombay College of Pharmacy, Kalina, Mumbai-400098, India.**

**Introduction:**

The pharmacodynamic effects of drug interactions are often unpredictable, because the pathophysiology of each patient with a given clinical abnormality is quite variable. Combination therapy has proved useful in treatment of many cardiovascular disorders caused due to diabetes. The pharmacotherapy for cardiovascular disease is generally not been designed with the diabetic population in mind, yet there is evidence that diabetic vasculature differs from non-diabetic vasculature. In view of these observations, it was felt that the nature as well as extent of drug interaction may differ in normal and diabetic condition.

**Aim:**

The aim of present investigation was to compare the interaction of combination of forskolin, with other anti-hypertensive agents on isolated rat thoracic aorta in normal and diabetic rat.

**Methods:**

Isometric tension recording of isolated aortic rings was done for the combination of forskolin with prazosin and that of forskolin with nifedipine. Concentration response curves were constructed and percentage relaxation was measured. The drug interactions were evaluated with isobolographic analysis.

**Results:**

Combination of forskolin and prazosin showed additive effect in normal rat thoracic aorta (Z_add_≈Z_mix_) (1.815nM≈2.11nM) while it showed synergism in diabetic rat thoracic aorta (Z_add_>Z_mix_) (1.02nM>0.688nM), while that of forskolin and nifedipine showed additive effect in normal rat thoracic aorta (Z_add_≈Z_mix_) (28.06nM.31.38nM) and synergistic effect in diabetic rat thoracic aorta (Z_add_>Z_mix_) (13.1nM>8.518nM).

**Conclusion:**

Forskolin when combined with other anti-hypertensive agents can be used at lower doses for effective anti-hypertensive therapy in a diabetic condition and has potential clinical benefit.

**250**

**Hesperidin protects against experimentally–induced myocardial ischemia reperfusion injury in rats**

Gujjar V, Gandhi C, Balaraman R

**Faculty of Technology and Engineering, The M. S. University of Baorda, Vadodara, India.**

**Aim:**

Among the heart diseases, ischemia and reperfusion induced arrhythmias contribute to episodes of sudden death. Cardiac arrhythmias during ischemia reperfusion (I/R) are believed to be related to oxidative stress. Therefore, aim of this study, was to examine whether treatment with Hesperidin alleviate arrhythmias and infarct size in experimentally -induced myocardial I/R injury using an *in vivo* rat model.

**Materials and Methods:**

In the study animals were divided in to sham, I/R control, I/R+HSP (Hesperidin treated I/R control group), I/R+Vit.E (vitamin E treated I/R control group) groups. Homodynamic parameters, markers of inflammation, biomarkers of oxidative stress, tissue nitrite level, DNA fragmentation and infarct size of the heart were estimated for each animal.

**Results:**

Animals subjected to I/R showed significant decrease in tissue nitrite, biomarkers of oxidative stress level and significant increase in inflammation and myocardial cell apoptosis and necrosis as compared to sham-operated group. In Hesperidin treated I/R control group, there was a significant increase in tissue nitrite, biomarkers of oxidative stress level and reduction in inflammation and apoptosis as compared to I/R control group.

**Conclusion:**

Protecting effect of Hesperidin in I/R is due to reduction in inflammation and oxidative stress.

**251**

**Effect of conventional antihypertensives on hypolipidemic action of garlic in rats**

Kumar PSR^1^, Asdaq SMB^1^, Inamdar MN^2^

**^1^Krupanidhi College of Pharmacy, Bangalore, India; ^2^Al-Ameen College of Pharmacy, Bangalore, India.**

Concurrent administration of conventional antihypertensives such as propranolol (PRO), hydrochlorthiazide (HYD) and captopril (CAP) with garlic homogenate (GH) may influence the activity of each other. The medicinal value of garlic is best known for its lipid lowering and antiatherogenic effects. The objective of the present study was to determine the possible alteration in hypolipidemic actions of GH in presence of PRO, HYD and CAP. Albino rats fed with either normal fat diet (NFD) or high fat diet (HFD) were treated with GH at three different doses of 125 mg/kg, 250 mg/kg and 500 mg/kg orally for 30 days or different doses of GH along with PRO (10 mg/kg, p.o), HYD (10 mg/kg, p.o) and CAP (30 mg/kg, p.o) during the last 7 days of GH treatment. At the end of treatment period, total cholesterol (TC), LDL-cholesterol, triglyceride (TG) and HDL-cholesterol were measured in serum and antiatherogenic index was calculated. The result showed that moderate and high doses of GH possess potential antiatherosclerotic property that was significantly attenuated by PRO and HYD. However, CAP augmented GH antihyperlipidemic activity. It was concluded that administration of PRO and HYD decrease the hypolipidemic effect of GH and administration of GH along with CAP augments its hypolipidemic effect in rats.

**252**

**Effects of some animal bile secretions on the normal heart**

Prasad N, Uday Kiran V, Murali G, Praveen B, Ramakrishna D, Prabhakara MC

**Kakatiya University, Warangal, India.**

**Objective:**

Based on the fact that the animal ‘PITTA’ or Bile has different medicinal properties and therapeutic uses, we have studied the effect of sheep and goat biles on the activity of heart.

**Methods:**

The present study was aimed at investigating the effect of bile on the isolated heart and to study the influence of bile on the action of some commonly used agonists like isoprenaline, phenylephrine. Some animal bile secretions mainly caused bradycardia in frog *Rana tigerina*. Frog heart was isolated and the effect of bile secretions in different concentrations was studied by syme’s technique.

**Results and Discussion:**

The disturbance in ventricular rhythm was observed prior to than that of atria. Rhythm was specially disturbed at higher doses causing bizarre pattern. Force of contraction of the heart also decreased with higher dose of the sheep and goat bile. The results suggest that the bile of selected animals has some beneficial effect on heart rate modulating the rate, rhythm and force of contraction positively but very high doses may exert nondesirable effects as well. Sheep and goat bile at 50 times dilution in frog Ringer’s solution elicited arrhythmogenic activity.

**Conclusion:**

We can conclude that the sheep and goat bile secretions can be used as arrhythmogenic agents in isolated frog heart preparations and may be useful for screening of antiarrhythmic agents.

**253**

**Cardioprotective interaction of captopril with garlic in isoproterenol induced myocardial infarction in rat**

Devkar SS^1^, Asdaq SMB^1^, Inamdar MN^2^, Premkumar N^1^

**^1^Krupanidhi College of Pharmacy, Bangalore, India; ^2^Al-Ameen College of Pharmacy, Bangalore, India.**

**Introduction:**

Garlic (*Allium sativum*) is reported to possess cardioprotective, antioxidant, antiarrhythmic and antiatherogenic properties. Concurrent administration of garlic with conventional antihypertensives such as captopril (CAP) during myocardial infarction (MI) may influence activity of each other. Thus the present study was carried out to explore possible cardioprotective interaction of garlic homogenate (GH) with CAP in isoproterenol (ISO) induced MI in rats.

**Methods:**

Female Wistar albino rats were treated with GH at three different doses of 125; 250 and 500 mg/ kg orally for 30 days and CAP (30 mg/kg, *p.o*.) was incorporated in the interactive groups during the last seven days of GH treatment. Myocardial damage was induced by administration of ISO (150 mg/kg, *s.c*.) for 2 consecutive days. Blood was withdrawn 48 hrs after the first dose of ISO and serum was separated. Heart tissue homogenate (HTH) of the excised heart was prepared. Both serum and HTH were used for estimating endogenous biomarkers such as lactate dehydrogenase (LDH) and creatine phosphokinase isoenzyme (CK-MB) as well as biological antioxidants like superoxide dismutase (SOD) and catalase (CAT) activities. Histopathological studies were carried out subsequently using hematoxylin and eosin stain.

**Results and Conclusion:**

The CAP and GH 250 mg/ kg was found to dislodge the effect of ISO on SOD and CAT and retained the activities of LDH and CK-MB. Incorporation of CAP during GH treatment provided further protection to myocardium from injury. However, higher dose of GH alone or with CAP failed to prevent damaging effects of ISO. Histopathological determinations confirmed biochemical findings.

**254**

**Effect of myricetin on DOCA-salt induced hypertension in wistar rats**

Jaiswal BS, Borde PP, Mohan M, Kasture SB

**MGV–s Pharmacy College Nashik-03, India.**

**Introduction and Objective:**

DOCA salt a - mineralocorticoid induced hypertension is dependent on salt and water. The objective of the study was to determine the effect of myricetin obtained from *Vitis vinifera* Linn. (Vitaceae) on blood pressure and oxidative stress in DOCA-salt induced hypertensive rats.

**Materials and Methods:**

The unilateral nephrectomized female rats were divided into 6 groups of 5 rats each. Group I received daily injection of 0.1ml of sterilized cotton seed oil subcutaneously, Group II received myricetin (100 mg/kg/day, p.o), Group III received myricetin (300 mg/kg/day, p.o), and Group IV received DOCA (25 mg/kg /week, s.c.) dissolved in sterilized cotton seed oil, Group V received DOCA, myricetin (100) and Group VI received DOCA, myricetin (300) for 4 weeks and all groups received 1% saline and 0.2% KCl *ad libitum* as drinking water. After the treatment schedule vascular reactivity to various catecholamines (Adr, NA, PE, Ang-II, 5-HT) were determined using Powerlab/4sp (ADInstrument, Australia) and antioxidant enzyme Levels of SOD, CAT, GSH and TBARS were determined from heart tissue in various groups. CCRC of 5-HT and Ang-II were obtained by using isolated rat fundus strip and isolated rat ascending colon strip.

**Result:**

Myricetin treatment significantly (*P*<0.05) reduced systolic blood pressure, vascular reactivity changes and reversed DOCA induced increase in heart rate and significantly (*P*<0.05) increased antioxidant enzyme levels and significantly (*P*<0.05) decreased TBARS level. CCRC of 5-HT and Ang-II were shifted to right in myricetin treated groups.

**Conclusion:**

Chronic administration of myricetin reduces elevated blood pressure in DOCA-salt hypertensive model possibly through its potent antioxidant action.

**255**

**Elevated glucose transcriptionally upregulates protease-activated receptor-4 (PAR-4) thrombin receptors in human vascular smooth muscle cells**

Dangwal S, Schrör K, Rosenkranz AC

**Institut für Pharmakologie & Klinische Pharmakologie, Universitätsklinikum, Henrich Heine University Düsseldorf, Germany.**

**Objective:**

Diabetes promotes inflammation, thrombosis and vascular remodeling. The clotting factor thrombin stimulates smooth muscle cell (SMC) mitogenesis via protease-activated receptors (PAR-1, PAR-3, PAR-4). We investigated if elevated glucose transcriptionally regulates PARs in human vascular SMC.

**Methods:**

Human saphenous vein SMC maintained in normal glucose (5.5 mM) were stimulated with high glucose (25 mM) ± study drugs. PAR mRNA, protein expression and promoter activity were determined by real-time PCR, western blotting/immunofluorescence and luciferase reporter assay.

**Results:**

PAR-1 and PAR-3 were not regulated by high glucose. PAR-4 mRNA was induced 3-fold within 1.5h (n=7, *P*<0.05) and sustained significantly to 96h, accompanied by increased protein expression (n=5, *P*<0.05). PAR-4 promoter activity was also induced at 6-48h (n=4, *P*<0.05), indicating regulation at transcriptional level. Accordingly, stimulatory effect of PAR-4 activating peptide (GYPGQV, 200microM) on TNFalpha expression was enhanced in SMC pretreated (48h) with high glucose. Since diabetes is reported to induce cyclooxygenase-2, we investigated if prostaglandins could regulate PAR-4. Exogenous PGE2 (1microM) or the stable prostacyclin analogue cicaprost (10nM) suppressed PAR-4 mRNA at 6-24h (n=5, *P*<0.05). Similar downregulation by the adenylate cyclase activator forskolin (10 microM) and the presence of a CREB site in the PAR-4 promoter implicates cyclic AMP as a possible counter-regulatory signal to transcriptionally control PAR-4.

**Conclusion:**

Elevated glucose induces a rapid and sustained induction of PAR-4 in human vascular SMC, while vasodilatory prostaglandins have an opposite effect. Since PAR-4 contributes to the mitogenic actions of thrombin, such transcriptional regulation might be relevant for the enhanced vascular remodeling in diabetes.

**256**

**Mechanism of ischaemic and CCPA preconditioning in hyperlipidaemic rat hearts**

Bhangu G, Reddy BV, Singh M

**Department of Pharmacology, I.S.F. College of Pharmacy, Moga- 142 001, Punjab, India.**

**Objective:**

The present study has been designed to investigate the mechanism of ischaemic and CCPA preconditioning in hyperlipidaemic rat heart.

**Materials and Methods:**

Experimental hyperlipidaemia developed by administering high fat diet, was confirmed by estimating serum levels of total cholesterol, LDL, HDL, VLDL and TGs. Isolated Langendroff’s perfused rat heart was subjected to ischemic preconditioning (IPC: four episodes of ischemia or CCPA and reperfusion of 5 min each). Global ischemia (30 min) followed by reperfusion (120 min) was employed to produce myocardial ischaemia and reperfusion (I/R) injury. The extent of myocardial injury was assessed by measuring myocardial infarct size using TTC staining and estimation of LDH and CK-MB in the coronary effluents.

**Results:**

Both IPC and CCPA preconditioning failed to reduce the I/R-induced increase in myocardial infarct size, LDH and CK-MB release in hyperlipidaemic rat hearts. Further, pretreatment with diadzein (2 mg kg^−1^, s.c., 1 week), a caveolin inhibitor, markedly attenuated the effect of IPC in hyperlipidaemic rat hearts. However, pretreatment with diadzein (2 mg kg^−1^, s.c., 1 week), in CCPA preconditioning protected the isolated hyperlipidaemic rat hearts by significantly attenuating the myocardial infarct size, release of LDH and CK-MB in coronary effluent.

**Conclusion:**

It may be concluded that caveolin inhibition may be responsible for the reversal of hyperlipidaemia induced attenuation of cardioprotective effect of CCPA preconditioning.

**257**

**Antagonistic role of quercetin against cardiotoxicity and impaired anti-oxidation induced by doxorubin in Swiss albino mice**

Dey S, Jain P, Kumar MNS, Vadivelan R, Antony AS, Elango K, Suresh B,

**JSS Mahavidyapeetha, Mysore, India; JSS College of Pharmacy, Ooty, India.**

Radio and/or chemotherapy are known to be the two major modalities in cancer treatment. Drugs exhibiting effective control of cancer tumors showed to exert cumulative toxicity that limits their clinical use or disrupts the continuity of treatment. Among several hypothesis put forward to explain the mechanism of radiation and chemical toxicity is the one involving the generation of free radicals. In this investigation 30 male Swiss Albino mice (20- 25g) were categorized into five groups G1 – G5 (6 in each group) and were provided with standard pellet diet and water *ad libitum*. Group I was kept as solvent control with 0.9% NaCl intraperitoneally. Group II – Quercetin (40mg/kg/ip), Group III – Doxorubicin (15mg/kg/ip), Group IV – Doxorubicin (15mg/kg/ip) + Quercetin (10mg/kg/ip), Group V – Doxorubicin (15mg/kg/ip) + Quercetin (20mg/kg/ip). Lactate Dehydrogenase (LDH), Creatinine Phosphokinase (CPK), Reduced Glutathione (GSH) levels in the serum after 11 days was assayed as sensitive parameter for cardiac dysfunction and anti – oxidant properties of Quercetin. Results demonstrated that Doxorubicin induces cardiotoxicity as evidenced by an increase in LDH, CPK, GSH levels. The results indicated that Quercetin possess marked dose dependent cardioprotective activity due to its anti oxidant property and thus have a promising role to play in the treatment of Doxorubicin induced cardiotoxicity. The application of Quercetin as an adjuvant or as a food additive (soya bean, tea, liquorice) during chemotherapy or the treatment of diseases linked to oxidative stress may therefore be beneficial.

**258**

**Human umbilical cord blood cells (hUCBCs) improves myocardial function in doxorubicin-induced cardiomyopathy in wistar albino rats**

Panigrahi A^1^, Das A^2^, Y Rojaramani^1^, Pradhan S^1^, Maharana C S^1^

**^1^MKCG Medical College, Berhampur, India; ^2^Kanak Manjari Institute of Pharmaceutical Sciences, Rourkela, India.**

**Introduction:**

Cardiomyopathy is the deterioration of the function of the myocardium and often at risk of arrhythmia or sudden cardiac death. Human umbilical cord blood is rich in mesenchymal progenitor cells. So it could be an optimal source for cellular repair of heart. The present study was undertaken to find out the role of human umbilical cord blood cells (hUCBCs) on doxorubicin (DOX)-induced cardiomyopathy.

**Materials and Methods:**

Cardiomyopathy was induced in rats by administering doxorubicin 2.5 mg/kg in normal saline (i.p.) in six equal injections in a period of 2 weeks. Then hUCBCs given to the animals intraperitoneally for 3 days (3×10^6^ cells in 0.5 ml in normal saline). Normal and Dox control group received saline and doxorubicin only for 2 weeks respectively. All the animals were observed for 4 weeks and evaluated for biochemical parameters (LDH and CK), ECG, echocardiography and histopathological changes; the mortality rate was determined in each group. (n=10).

**Results:**

There was significant reduction in LDH and CK level in hUCBCs treated animals when compared to the Dox control group. The elevated ST segment seen in doxorubicin treated animals was normalized in hUCBCs treated group. Echocardiography demonstrated an improvement in fractional shortening (FS). Histopathological changes consisted of patchy necrosis, vacuolated hypertrophied myocardial fibers and fibrosis in the doxorubicin controls with significant improvement in the hUCBCs treated group. hUCBCs treatment reduced mortality to 20%.

**Conclusion:**

Cord blood cells may be a potential source for cell-based therapy for doxorubicininduced cardiomyopathy.

**259**

**Cardiotoxic effects of naproxen in doxorubicininduced cardiomyopathy in rats**

Ahmad R, Pillai KK, Dubey K

**Hamdard University, Hamdard Nagar, New Delhi-110062, India.**

**Introduction:**

In the repercussion of the heated dispute on cyclooxygenase -2 (COX-2) selective nonsteroidal anti-inflammatory drugs (NSAIDs) led to the national and international withdrawal of several of the recently introduced coxibs. Further debate and research has highlighted risks of the classical NSAIDs too. There is much controversy about the cardiovascular safety of a non selective NSAID naproxen and its possible cardioprotective effect. The study was undertaken to determine the cardiovascular effects of naproxen on doxorubicin induced cardiomyopathy in rats.

**Methods:**

Male albino rats received a single i.p. injection of normal saline (normal control group) and doxorubicin (DOX) 15mg/kg (toxic control group). Naproxen (NAP) was administered alone (50mg/kg/day, p.o.) and in combination with DOX and DOX + trimetazidine (TMZ) (10mg/ kg/day, p.o.) for 5 days after 24 hrs of DOX treatment. Doxorubicininduced cardiomyopathy was assessed in terms of increased activities of cardiac marker enzymes (lactate dehydrogenase, tissue thiobarbituric acid reactive substances) and decreased activities of antioxidant enzymes (glutathione, superoxide dismutase and catalase), followed by transmission electron microscopy of the cardiac tissue.

**Results:**

Doxorubicin significantly increased oxidative stress as evidenced by increased cardiac marker enzymes and decreased antioxidant enzymes. Both biochemical and electron microscopic studies revealed that naproxen itself was cardiotoxic and aggravated doxorubicin-induced cardiomyopathy and abolished the protective effect of trimetazidine.

**Conclusions:**

Present study indicates that naproxen has the potential to worsen the situation in cardiovascular compromised patients. So, it should be used cautiously in heart patients.

**260**

**Comparative evaluation of rosiglitazone and pioglitazone on doxorubicin induced cardiomyopathy in rats**

Saraogi P, Dubey K, Pillai K K

**Hamdard University, New Delhi, India.**

PPAR gamma receptor agonists are widely used drugs in the management of type 2 diabetes. Recently cardiovascular safety of Rosiglitazone has been questioned. Therefore present study was designed to evaluate weather these drugs aggravate or attenuate the doxorubicin induced cardiomyopathy in rats. In this study 48 rats were used (weighing between 250-300g) and divided in to 6 groups (8 rats in each group). First group was control group (given 0.5%C.M.C.) and second group was toxic control received 15mg/kg of doxorubicin i.p. other groups received treatment with Rosiglitazone(5mg/kg) and Pioglitazone(10mg/kg) in combination with doxorubicin for 14 days and doxorubicin was administered on 10^th^ day. Similarly the per se effect of these drugs was also evaluated. Following biochemical parameters were observed after completion of duration of treatment LDH, total cholesterol level, triglycerides, LDL, VLDL, HDL. Histology and transmission electron microscopy was also carried out of rat cardiac tissue. Both Rosiglitazone and Pioglitazone raised the serum LDH level significantly and showed cardiotoxic effect on rat myocardium. Rosiglitazone significantly worsened the cholesterol profile raised the level of LDL however Pioglitazone showed a shift towards benefit it raised the level of HDL. Histopathology and transmission electron microscopy showed that these caused extensive damage to rat myocardium and showed aggravation of doxorubicin induced cardiomyopathy. TZDs agonists Rosiglitazone and Pioglitazone found to be cardiotoxic in this rat model doxorubicin induced cardiomyopathy.

**261**

**Novel regulation of glycogen synthase kinase-3β activity by matrix metalloproteinase-2**

Kandasamy AD, Cho WJ, Schulz R

**Cardiovascular Research Group, University of Alberta, Edmonton, Alberta, Canada.**

Matrix metalloproteinase (MMP)-2 is localized to the sarcomere of cardiomyocytes and contributes to myocardial ischemia-reperfusion injury by degrading sarcomeric proteins troponin I and myosin light chain 1 and cytoskeletal (alpha-actinin) proteins. Oxidative stress mediates its earliest cascade of damaging effects through activation of MMP-2 within the cardiomyocyte and subsequent proteolysis of susceptible intracellular targets. Glycogen synthase kinase (GSK)- 3beta is a multitasking serine/threonine kinase involved in growth, death and metabolism and is susceptible to proteolytic cleavage. We determined whether GSK-3beta is a proteolytic substrate of MMP-2 and how this cleavage affects its kinase activity. Incubation of hrMMP-2 and hrGSK-3beta at 30°C resulted in a time- and concentration-dependant cleavage of GSK-3β as evidenced using SDS-PAGE as lower molecular weight fragments appearing around 30 kDa, detected by immunoblotting and silver stain. GSK-3beta kinase activity was significantly increased (2-3 fold) upon incubation with MMP-2. MMP-2 mediated enhanced kinase activity was prevented by serine specific dephosphorylation of GSK-3beta with lambda-phosphatase. H_2_O_2_ treated H9c2 cardiomyoblasts showed increased levels and activity of MMP-2 accompanied by a significant reduction in GSK-3beta protein levels and increased kinase activity. Co-localization studies using double-immunofluorescent labeling in rat ventricular sections revealed that GSK-3beta and MMP-2 are co-localized at the sarcomere and Z-discs. These data indicate that GSK-3beta is a target of MMP-2 and that its cleavage by MMP-2 enhances GSK-3beta kinase activity. GSK-3beta dephosphorylation studies suggest that MMP-2 may cleave off a peptide containing the inhibitory serine-9 residue. Hence, this MMP-2 mediated augmentation of GSK-3beta kinase activity may contribute to cardiac dysfunction as a result of myocardial oxidative stress injury.

**262**

**Protective effects of *Tribulus terrestris* against myocardial injury induced by isoproterenol in rats: biochemical and histological findings**

Ray I, Maheshwari U

**MGM Medical College, Navi Mumbai, India.**

**Introduction:**

The present study was designed to investigate whether *Tribulus terrestris* (Tt), a natural herb, would attenuate the acute myocardial infarction in isoproterenol (ISP)-treated rat model via maintaining activities of endogenous antioxidant enzymes.

**Methods:**

The ISP model of myocardial necrosis was used for evaluation of therapeutic intervention of Tt on the extent of jeopardized myocardium and evolution of infarction in ISP administered rats. Cardiac marker enzyme: Creatinine phosphokinase(CPK) and antioxidative parameters: Glutathione (GSH), Thiobarbituric acid reactive substances (TBARS), Catalase (CAT) and Superoxide dismutase (SOD) of heart tissues were measured. Histopathological examination of heart tissues was also performed.

**Results:**

Induction of rats with ISP (85 mg/ kg) subcutaneously on 20^th^ and 21^st^ day resulted in significant cardiac necrosis, decline in antioxidant status and elevation in lipid peroxidation. Oral administration of Tt (2.5, 5 and 10 mg/ kg, respectively) to healthy experimental animals (i.e. animals without any myocardial pathologic challenge viz. ISP) for 21 days significantly enhanced the basal myocardial levels of GSH (*P*<0.05), CAT (*P*<0.05) activity as compared to the sham group. Subsequent to ISP induced myocardial injury, Tt (2.5 and 10 mg/kg) pre-treatment demonstrated significant mitigating effects on several myocardial injury induced biochemical {GSH (*P*<0.05), TBARS (*P*<0.05), CPK (*P*<0.05)} and histopathological perturbations. Tt (2.5 mg/kg) was found to be the optimum cardioprotective dose.

**Conclusion:**

The results indicate that chronic Tt administration causes myocardial adaptation by augmenting endogenous antioxidants and protects rat hearts from oxidative stress associated with ISP induced myocardial injury.

**263**

**Chronic oral administration of *Ocimum sanctum Linn*. a phytoadaptogen, preserves chronic restraint stress induced protection against myocardial ischemia-reperfusion injury in rats**

Enjamoori Rajesh^1^, Kumar Santosh^1^, Jaiswal Amardeep^1^, Sood Subeena^2^, Dinda Amit K^3^, Maulik Subir K^1^

**^1^Department of Pharmacology, All India Institute of Medical Sciences, New Delhi, India; ^2^Department of Molecular Physiology and Biophysics, Baylor College of Medicine, Houston, Texas, USA; ^3^Department of Pathology, All India Institute of medical Sciences, New Delhi, India.**

**Objective:**

To study effects of *Ocimum sanctum Linn*. in myocardial ischemia reperfusion injury following chronic restraint stress in rats.

**Materials and Methods:**

Male Wistar rats were administered with hydro-alcoholic extract of *Ocimum sanctum* (Os; 100 mg/kg; orally for 21 days) alone or along with chronic restraint stress (CRS; 6 h/ day for 21 days). This was followed by ischemia reperfusion injury (IR; 20 min ischemia followed by 40 min reperfusion) of isolated hearts from different groups *viz*. Control, Os, CRS and CRS + IR, using Langendorff apparatus.

**Results:**

In Control IR, there was increased oxidative stress, as evidenced by significant increase in myocardial TBARS (20%; *P*<0.05) and depletion of myocardial endogenous antioxidants, SOD (40%; *P*<0.01), catalase (12%), GSH (35%; *P*<0.01) and GPx (16%; *P*<0.05) as compared to normal Control. However, CRS rats were resistant to IR induced cardiac changes, in terms of increased endogenous antioxidants (myocardial GSH level, SOD and catalase activities) as compared to Control IR. *O. sanctum per se* showed protective effect against IR injury, with significant prevention of rise in myocardial TBARS (36%; *P*<0.01) and fall in GSH level (48%; *P*<0.001), along with preservation of SOD (102% *P*<0.001) and GPx (17%; *P*<0.05) activities as compared to Control IR. Moreover, IR injury was significantly prevented in CRS rats fed with *O. sanctum*, with prevention *P*<0.01 of rise in myocardial TBARS and fall in GSH level *P*<0.05; along with no significant change in myocardial of SOD (*P*<0.01) and GPx (*P*<0.01) activities as compared to Control IR.

**Conclusion:**

The present study highlights the holistic action of *Ocimum sanctum* as an adaptogen.

